# The REDUCE Intervention: The Development of a Person‐Centred Cognitive Behavioural Intervention to Improve Ulcer Outcomes in People at Risk of Diabetic Foot Ulceration

**DOI:** 10.1111/hex.70434

**Published:** 2025-09-18

**Authors:** Kavita Vedhara, Debbie Brewin, Fran Game, Christina Sheehan, Kieran Ayling, Kat Bradbury, Joanna Slodkowska‐Barabasz, Judith Joseph, Ruth Hart, Natasha Mitchell, Julia Lawton, Trudie Chalder

**Affiliations:** ^1^ School of Psychology Cardiff University Cardiff UK; ^2^ Institute of Psychiatry, Psychology & Neuroscience (IoPPN) King's College London London UK; ^3^ Royal Derby Hospital University Hospitals of Derby and Burton Foundation Trust Derby UK; ^4^ Centre for Academic Primary Care, School of Medicine University of Nottingham Nottingham UK; ^5^ School of Psychology University of Southampton Southampton UK; ^6^ School of Primary Care, Population Sciences and Medical Education University of Southampton Southampton UK; ^7^ Usher Institute University of Edinburgh Edinburgh UK; ^8^ York Trials Unit, Department of Health Sciences University of York York UK

**Keywords:** behaviour, complex interventions, diabetes, diabetic foot ulcers, person‐based approach, pilot trial, think‐aloud

## Abstract

**Introduction:**

Diabetic foot ulcers (DFUs) affect approximately one‐quarter of people living with diabetes. They are chronic, recur frequently and are associated with significant psychological distress and behavioural challenges. The REDUCE intervention is a person‐centred, cognitive behavioural intervention designed to reduce the risk of DFU recurrence and support ulcer healing. Here, we describe the iterative development and optimisation of REDUCE, from its inception as a group‐based intervention to an individually tailored intervention delivered via video call or telephone. We outline key stages of the intervention development, including the integration and modification of a digital maintenance intervention (DMI) designed to support long‐term behaviour change and a mixed‐methods external pilot trial which informed a full‐scale clinical and cost‐effectiveness trial.

**Methods:**

After initial development, the DMI was the subject of nine ‘think‐aloud’ interviews with patient and public contributors. We conducted an external pilot randomised controlled trial, involving 20 patients with recently healed DFUs randomised in a 2:1 ratio (REDUCE + Usual Care vs. Usual Care only). Data collection included patient‐reported outcome measures (baseline and 6 weeks and 3 months post‐randomisation) and qualitative interviews with participants and facilitators.

**Results:**

Think‐aloud interviews informed key refinements to the DMI to enhance usability and engagement. The pilot trial demonstrated high acceptability of the intervention format and delivery. Patient‐reported outcomes suggested positive trends in psychological well‐being, footcare behaviours and mood among intervention participants. Qualitative findings highlighted the value of individualised delivery, the importance of facilitator support and varied engagement with the DMI. These insights informed further refinements to REDUCE ahead of a full‐scale effectiveness trial.

**Conclusion:**

We provide a comprehensive account of the evolution of the REDUCE intervention and share broader learnings regarding the development of complex behavioural health interventions. The example of REDUCE highlights the value of iterative, multidisciplinary methods and patient involvement in intervention design and offers practical insights for designing digital and remote health interventions.

**Patient or Public Contribution:**

Patient and public contributors were involved throughout the research described in this manuscript. Key areas of involvement included co‐creation of all patient‐facing materials, intervention development and informing trial methods.

## 1 Background

Current prevalence data suggest that one in 10 people in the United Kingdom has diabetes [1]. Diabetic foot ulcers (DFUs), defined as a full‐thickness penetration of the dermis of the foot, are a common, chronic and costly complication of the disease. It is estimated that between 19% and 34% of people with diabetes will get a DFU in their lifetime [2], less than half of whom will be ulcer‐free after 6 months of treatment [3], and 80% of the 3000 major amputations in people with diabetes each year are preceded by DFUs [4]. The physical and emotional burden of ulceration is considerable: 32% of patients are depressed, which, in turn, is related to a threefold greater risk of mortality [5]. Indeed, the profound psychological sequelae of the disease and its treatment [3] led the National Institute for Health and Care Excellence (NICE) to advocate for the development of interventions that target psychological and behavioural risk factors [4]. REDUCE is such an intervention. It has been designed to reduce the risk of ulceration and promote healing if ulceration occurs. The target patient group is people with a history of ulceration, who are at the highest risk of future ulcerations [6]. The intervention is currently the subject of a multi‐centre randomised controlled trial (RCT) [https://www.isrctn.com/ISRCTN15570706]. Here, we provide a brief overview of the history of REDUCE and describe the iterative process by which the intervention has been optimised for evaluation in the RCT. Our aims are two‐fold. First, to document in detail the content of the intervention and the evidence that guided its development, which will be relevant to future evaluations of REDUCE and its implementation. Second, to highlight broader learnings regarding the development of complex health interventions.

## 2 The History of REDUCE

**Figure 1:**
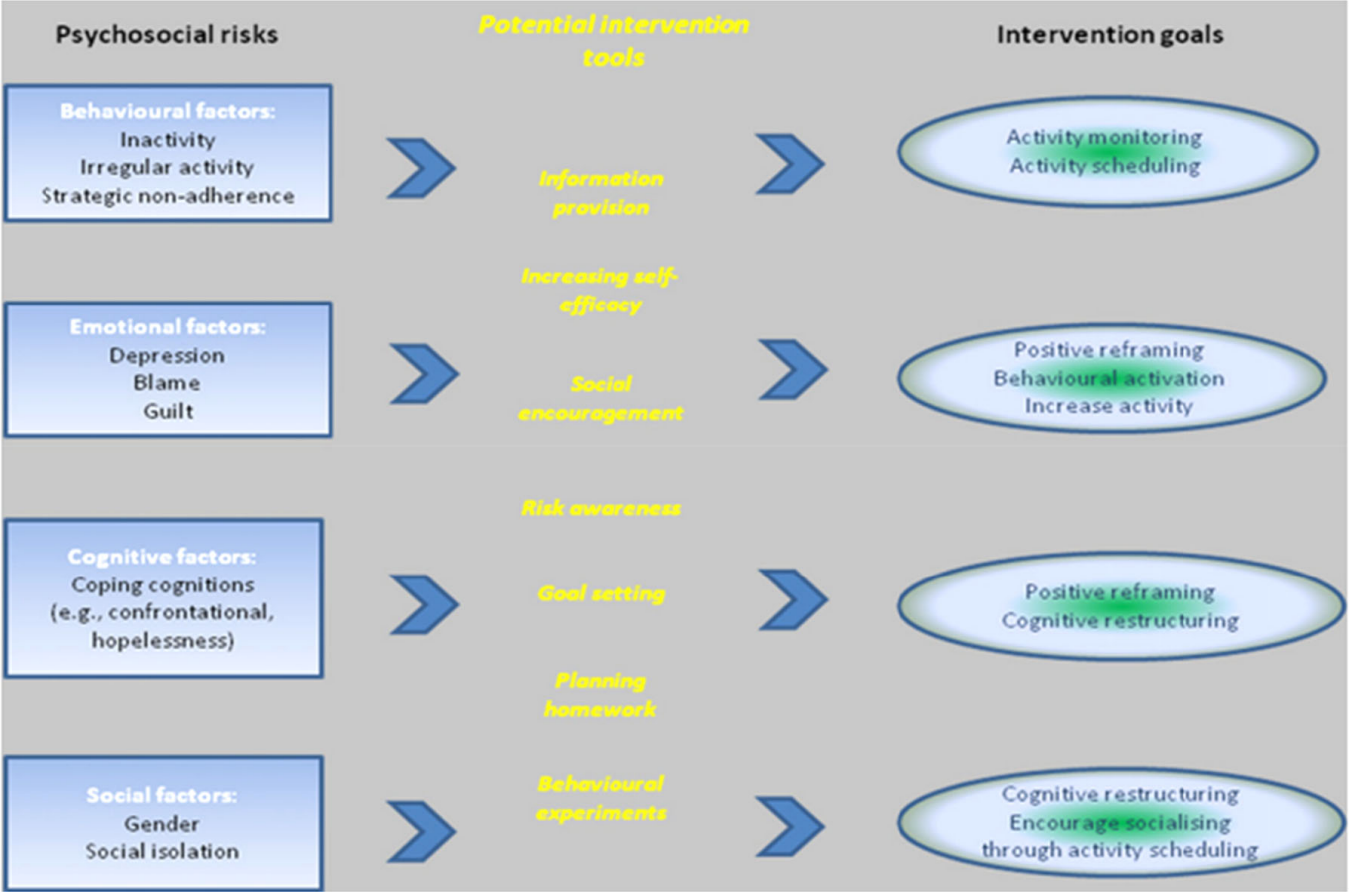
Original REDUCE logic model.

The first iteration of REDUCE has been described previously [7]. In brief, it was based on a cognitive‐behavioural therapeutic model targeting ‘psychosocial risk factors’ (i.e., behavioural, social, emotional and cognitive correlates of ulcer outcomes) evident in the literature at that time (see Figure 1). The model is based on the premise that behavioural, social, emotional and cognitive ‘systems’ are intimately connected. Although interactions between these systems can result in unhelpful patterns, changes in one area can also lead to improvements across others.

REDUCE was originally developed as a group‐based intervention consisting of two phases. The first (the ‘Initiation Phase’) was delivered over 10 weekly sessions and was designed to initiate changes in psychosocial risk factors. This was followed by a ‘Maintenance Phase’ consisting of three further sessions, held at 2‐month intervals, intended to maintain and sustain changes achieved in the first phase. The first evaluation of the intervention was a pilot RCT in which 15 people with diabetes and a history of ulceration were randomised in a 2:1 ratio to receive the intervention or usual care [7]. A qualitative evaluation provided evidence of acceptability and an early indication of sustained change up to 9 months post‐intervention in several of the psychosocial risk factors targeted in REDUCE. The findings also pointed to the need for revisions to the intervention, specifically reducing the ‘Initiation Phase’ to eight sessions and the ‘Maintenance Phase’ to two sessions.

**Figure 2:**
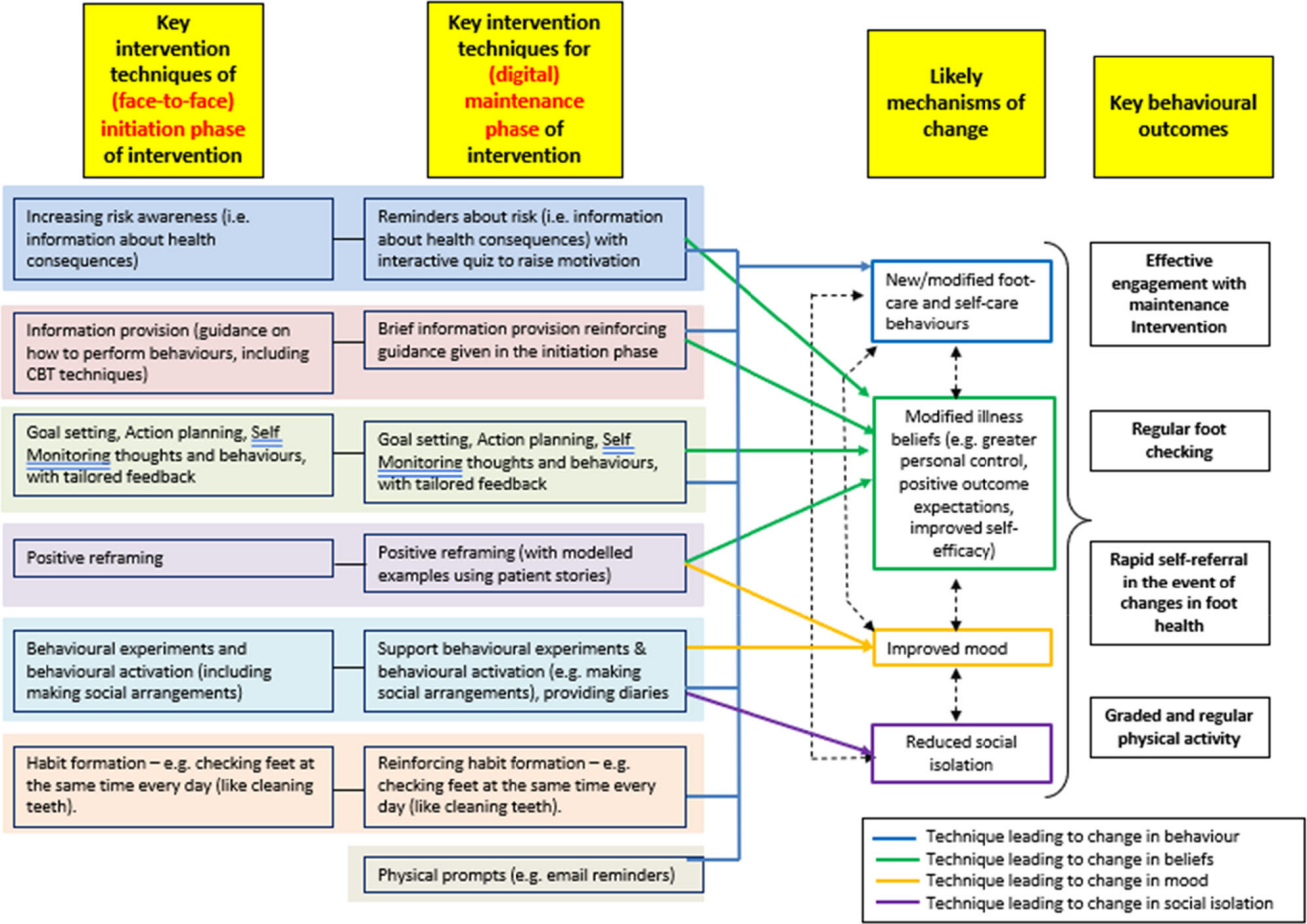
Revised logic model elaborating on intervention techniques deployed in the Initiation and Maintenance Phases of REDUCE.

Following this preliminary work, we explored enhancing the effectiveness of REDUCE by modifying the ‘Maintenance Phase’ to offer longer‐term support via a digital platform. Patient and Public Involvement and Engagement (PPIE) discussions at the time also supported this innovation. The rationale was to provide patients with support ‘on‐demand’, rather than being restricted to a limited number of stand‐alone sessions, while also mitigating potential costs of the intervention [8]. The methods used to develop a blueprint for the digital maintenance intervention (DMI) have been reported previously [9]. In brief, intervention planning followed established theory‐based, evidence‐based and person‐based approaches [10, 11, 12, 13] and was predicated on a scoping review of the literature to incorporate new evidence that had emerged since the original intervention was developed, and in‐depth qualitative interviews with people with a history of ulceration (*n* = 20). Together, these led to further elaboration of the original logic model and a clearer articulation of four key outcomes which patients would be encouraged to focus on to achieve sustained reductions in their risk of re‐ulceration and improved ulcer healing: regular foot‐checking; rapid self‐referral in response to changes in foot health; graded and regular physical activity when ulcer‐free; and emotional self‐management (see Figure 2).

The DMI website was developed in accordance with this blueprint, following which we undertook rapid PPIE work to optimise the DMI's functionality (Stage 1), followed by an external pilot trial of both the ‘Initiation Phase’ and DMI (Stage 2). The methods and results pertaining to both are presented below.

## 3 Methods and Results

### 3.1 Stage 1: Optimising the Functionality of the REDUCE DMI

The REDUCE DMI (https://www.reducestudy.co.uk/) incorporated the content of the original REDUCE face‐to‐face maintenance sessions, enhanced further by the functionality available through a website. Key web‐enabled features included: individualisation of content (i.e., allowing patients to access content from the four target areas according to their individual needs); online goal‐setting and monitoring in relation to each of the four areas; tailored feedback based on goal achievement; opportunities for modelling through videos of patient stories; and motivational emails to support long‐term changes in the four areas (sent weekly for the first 2 months and fortnightly thereafter). Maintenance booklets were also developed, which mirrored the content of the DMI (except for videos, tailored feedback and scheduled motivational emails). This ensured that, in a future trial, participants unable or unwilling to access the website would still receive support for long‐term behaviour change.

Ethical approval was granted by the North of Scotland Research Ethics Committee (ref: 19NS1071), and the research sponsor was the University Hospitals of Derby and Burton NHS Foundation Trust. This stage of the research coincided with the early stages of the Covid‐19 pandemic, during which all non‐Covid research was limited, and restrictions on social contact meant face‐to‐face research with participants was not possible. For these reasons, we conducted our intervention optimisation work with a small group of PPIE members (*n* = 4) aged 50–62, who had previously had a DFU (male [*n* = 2] and female [*n* = 2]; White British [*n* = 3] and Asian [*n* = 1]).

We adopted a person‐based approach [11] using PPIE feedback to identify modifications necessary to enhance engagement and address barriers to usage and adherence. The person‐based approach has been used extensively in the creation of digital interventions [14, 15, 16]. In the present work, this involved a series of iterative one‐to‐one *think‐aloud* interviews designed to permit an exploration of PPIE members' perceptions of the website as they used it, along with open‐ended questions allowing for deeper discussion [11, 17, 18].

#### 3.1.1 Think‐Aloud Interviews

Each PPIE member was interviewed individually, with sessions audio‐recorded and transcribed. Members took part in 2–3 interviews each (total *n* = 9), conducted over approximately 12 h in total. Verbal consent to record the sessions was obtained at the start of each session. Sessions began with the participants being asked to view the REDUCE DMI and to ‘think out loud’ (i.e., verbalise their thoughts, likes and dislikes about each page, not limited to content, images, videos, design, format, layout, navigation and flow).

#### 3.1.2 Analysis

The Person‐Based Approach's pragmatic analysis methodology [19] involves tabulating positive and negative comments from transcribed interviews and listing intervention modifications based on this feedback. Within this process, modifications are prioritised based on whether they are likely to be critical to the usage of the website or engagement with offline behaviour changes targeted by the intervention, that is, the MoSCoW method (‘must‐have’, ‘should‐have’, ‘could‐have’ and ‘won't‐have’ [17]). Negative comments based on preferences unlikely to influence engagement (e.g., colour preferences) are not prioritised within this process. Analysis was conducted by two researchers (J.S.B. and J.J.) and discussed in detail with a third (K.B.), all of whom were experienced in qualitative research and the use of Person‐Based Approach methodology in optimising digital interventions.

## 4 Results

Throughout the think‐aloud process, PPIE members reported positive and negative comments about the DMI. Positive comments included the use of language considered easy‐to‐understand and encouraging, the overall appealing format, look and feel of the website pages and content (including images and videos), and clear, simple and helpful foot‐checking information. In addition, the inclusion of relatable patient stories which modelled changes in thoughts or behaviour and acknowledged that making behaviour changes can seem hard at first was valued. They also appreciated that the language used did not attribute blame to the patient but instead offered solutions to make things easier. However, PPIE members also highlighted potential challenges with the DMI. Examples of these are provided in Table 1 (classified according to the MoSCoW system) and any remedial actions taken.

**Table 1:** Examples of feedback on the DMI and actioned amendments.

Intervention/website feature	Think‐aloud feedback (quotes)	MoSCoW classification	Actions required and changes made
Accessibility across the website			
Navigation buttons are included within the website; however, additional buttons are required to aid usability given varying page lengths. Section content (in sub‐sections) within each section homepage can be accessed in any order.	‘*For me it would be nice to have a back button at the bottom … because as I am reading from top to bottom I am going to finish at the bottom of the page…’* [Reviewer1] ‘*…more of a flow … can go backwards and forwards … I would like to know that …I've seen everything on this page … and go forward until I get to the end and I will know that I've seen everything’* [Reviewer1]	Must have Should have	Additional buttons (identical to those used at the top of each page) added to the bottom of each page to enable forwards (‘next’) and backwards page movement. Page tunnelling introduced, whereby at the first visit for each section there is a defined pathway to ensure all content is provided in a particular order. At future accesses, the user can click on any of the sub‐sections and revisit accordingly.
Foot checking section			
Foot checking ‘how to’ guidance and videos. Importance of checking your feet every day.	‘*I think it would be nice to have pictures for each step that you have…. Look for changes in the colour of the skin on your feet, but then there are no more pictures’ [Reviewer4]* ‘*…if you mention the long term complications in terms of amputation and other. I don't know if it's right to say or not…. Because for some people … you need to open up their eyes in terms of showing what can happen if you don't’*	Won't have Won't have	No action required. The clinical team felt that these could be unhelpful or misleading as visual early signs can vary from person to person and it is more important to check feet regularly enough to know what is different in their feet, rather than working from a picture of what others' feet look like, which might have early signs of a problem that look quite different. Not actioned. Other reviewers disagreed. This was not changed as raising fear appraisals in this way might introduce disengagement and denial, rather than behavioural engagement in some users.
Getting help quickly (self‐referral) section			
Identifying key contacts and storing them on the website for future ease of access. Seeking help quickly when changes in foot/feet are observed.	‘*I've no idea what a key worker is … I don't recognise the word foot protection team’* [Reviewer1] ‘*It's not specific about how much the change has to be and … a lot of text that's in bold and it makes me feel a bit panicked as though you have to keep getting help’ [Reviewer3]*	Must have Won't have	Revised terminology to ‘details of people you contact when you have changes in your feet’ and ‘foot care specialists’ to encompass the range of healthcare professionals involved. Not actioned. Face‐to‐face sessions, which preceded the maintenance intervention, would help, guide and reassure participants before they arrive at the maintenance intervention.
Mood management section			
Ability to add and review values and activities, as well as how they relate to values and improve mood.	*You don't have a back button but that's fine … going back to the improving your mood page and you will see your answers and can come back so they can reflect further and make changes if they want to [Reviewer4]*	Must have	Buttons added to permit the user to return to look at the values and activities already entered.
Physical activity section			
Saving and updating physical activity and strength and balance goals from either a menu of options or free‐text to enable review of progress. Tailored strength and balance exercises: three levels of competency based on participant self‐assessment, including goals in minutes.	‘*But when you try and set a goal it doesn't press … I put in number of days and number of minutes and where it says next it's eventually happened but it took a while’ [Reviewer3]* ‘*I would expect a standard goal for people if you are doing something, an exercise…. Maybe I've missed it, maybe it wasn't clear … starting with 10 of these, 12 of those and 16 of those’ [Reviewer2]*	Should have Won't have	Additional text added to confirm goals are saved ‘Well done! You've set a new goal…’ Not actioned. Goals include the number of minutes for strength and balance exercises, not the number of times to complete the exercise.
Emails from the website			
Emails sent to motivate and encourage sustained behaviour change.	‘*[Receiving emails] can be annoying but at the same time if it is helping somebody then why not’ [Reviewer4]*	Must have	Ability to cancel all queued emails if requested.
My photos section			
*Area on the website for users to store photographs taken of their feet at various stages to help them identify early and ongoing changes in their feet and to be aware of their shape and any specific features*.	‘*I am reading the bit about pictures because that's interesting because I've got pictures of my feet … which … can be stored there which is good … if that is the case would … the medical professionals that I see be able to see them on [the] REDUCE website?’ [Reviewer1]*	Will not have	Due to confidentiality and data protection, photographs remain accessible only to the user. The website is not accessible to healthcare professionals treating the user.

### 4.1 Stage 2: REDUCE External Pilot RCT

Following development of the REDUCE DMI (and a hard‐copy booklet which mirrored the content of the DMI), a mixed‐methods external pilot RCT was conducted (ISRCTN15460422) to obtain feedback from patients and intervention facilitators on any aspects of the intervention that could be refined before conducting the main trial. This pilot trial represented the first evaluation of both phases of REDUCE following the original modification (i.e., the delivery of eight ‘Initiation Phase’ sessions [reduced from the original 10] and the provision of the ‘Maintenance Phase’ through the DMI and booklet [replacing the original three stand‐alone sessions]). Two additional changes to the mode of delivery were made, driven by emerging evidence, pragmatic considerations and the pandemic. The first was the decision to deliver the intervention one‐to‐one (rather than in groups). Although the original group intervention demonstrated promise, we foresaw problems when scaling up for a future trial and later implementation. This included challenges in recruiting sufficient numbers of recently healed patients in short time frames to form groups; the risk of attrition due to re‐ulceration before group sessions could begin; and logistical issues such as securing venues and arranging transport and access. Therefore, individual delivery was considered to offer a more pragmatic and personalised approach. Second, the intervention was delivered remotely (by telephone or video call) rather than in person. This was necessitated by the public health restrictions related to the Covid‐19 pandemic and supported by emerging evidence of comparable effectiveness of psychosocial interventions delivered via telephone or video call [20, 21].

The pilot trial was approved by the Wales Research Ethics Committee (REC ref: 21/WA/0110) and sponsored by the University Hospitals of Derby and Burton NHS Foundation Trust. The clinical findings and trial procedures are reported elsewhere [22] in line with CONSORT guidelines [23]. Here, we focus on patient‐reported outcome measures (PROMs) and intervention‐related findings from a nested qualitative study that played a key role in determining the need for future refinements to the intervention.

## 5 Methods

### 5.1 Recruitment and Eligibility

Potential participants who had a recently healed DFU were identified and screened by their clinical care team in specialist multidisciplinary diabetes foot clinics at participating NHS Trusts. To be included, participants had to have diabetes, be aged 18 or over, have two lower limbs, have a recently healed foot ulcer (healed for a minimum of 2 weeks) and be otherwise ulcer free. Participants were excluded if they had active Charcot neuro‐osteoarthropathy or cognitive, language or severe mental health impairments that could hinder engagement with the trial or intervention (e.g., psychosis). Participants were also excluded if they were participating in another study which could affect the outcomes of this study (e.g., wound‐healing medicinal product trial or another behavioural intervention study). Written informed consent was obtained by a suitably qualified and experienced research nurse, healthcare professional (HCP) or practitioner in accordance with Good Clinical Practice guidance. The sample size for this pilot RCT was not intended to assess intervention effectiveness, but to inform the design of a planned future clinical and cost‐effectiveness trial. We therefore sought to recruit a total of 15–20 participants, which was deemed sufficient to provide insight into intervention acceptability and trial processes.

### 5.2 Outcomes

Clinical outcomes (reported elsewhere [22]) were captured by blinded assessors at 4 months post‐randomisation (16 weeks) following review of clinical notes from primary, secondary and community care records. The primary outcome was ‘ulcer‐free days’ over the 16‐week follow‐up period. Secondary clinical outcomes included days to re‐ulceration (if applicable), number of ulcers, days in hospital, adverse events (AEs), serious adverse events (SAEs), amputations (major and minor), and mortality. PROMs (described below) were collected at baseline and 6 weeks and 3 months post‐randomisation.

### 5.3 Procedure

Following consent, patients provided baseline demographic data (e.g., date of birth, ethnicity, sex, etc.) and completed the PROMs summarised in Table 2 [23, 24, 25, 26, 27, 28, 29, 30, 31, 32, 33]. All PROMs were also completed at 6 weeks and 3 months post‐randomisation. Clinical information was obtained from clinical records. Following the completion of baseline data collection, participants were randomised in a 2:1 ratio to the intervention group to maximise the number receiving REDUCE.

**Table 2:** Patient‐reported outcome measures (PROMS) included in the pilot trial.

PROM	Citation	Internal reliability in the present study (*α*)
Brief Illness Perception Questionnaire (B‐IPQ)	Broadbent et al. 2006	—
Cognitive and Behavioural Responses Questionnaire (CBRQ)	Ryan et al. 2018	Fear Avoidance Subscale: 0.66 Damage Subscale: 0.73 Catastrophising Subscale: 0.75
Generalised Anxiety Questionnaire (GAD‐7)	Spitzer et al. 2006	0.94
Patient Health Questionnaire‐9 (PHQ‐9)	Kroenke et al. 2001	0.88
Mental Health Continuum Short Form (MHC‐SF)	Lamers et al. 2011	0.94
Nottingham Assessment of Functional Footcare (NAFF)	Lincoln et al. 2007	0.64
Scale of Positive and Negative Experience: Positive Mood Subscale (SPANE‐P)	Diener et al. 2010	0.95
Social Provisions Scale (SPS)	Cutrona and Russell, 1983	0.95
International Physical Activity Questionnaire—Elderly (IPAQ‐E)	Hurtig‐Wennlöf et al. 2010	—
Diabetic Foot‐Related Resource Use	Cullen et al. 2023	—

### 5.4 Nested Qualitative Study

Participants randomised to the intervention also consented to participate in the nested qualitative study. This consisted of interviews conducted at two time points: the first shortly after randomisation (‘baseline’ interview) and the second within a few weeks of the final Initiation Phase session (‘follow‐up’ interview). The HCPs who delivered the intervention (*N* = 3 ‘facilitators’) were also interviewed after delivery of their final intervention session. The interviews covered a range of issues. Here, we focus only on those relating to participants' and facilitators' experiences of receiving or delivering the intervention.

Interviews were semi‐structured and informed by topic guides. These provided scope for interviewees to raise issues they considered salient, including those unforeseen at the study outset. Interviews were conducted by an experienced qualitative researcher (R.I.H.), by video call or telephone, between June and December 2021. They averaged 1 h, were audio‐recorded and transcribed verbatim for analysis. Our analytical approach was pragmatic and focused on producing low‐inference descriptions of experiences and perspectives of direct practical relevance to the study aims [34]. Using the technique of ‘constant comparison’ [35], two experienced qualitative researchers identified, refined and detailed a set of descriptive themes [36]. These reflected both a priori concerns and emergent issues and were documented in a series of analytical reports, from which the findings reported below are drawn.

### 5.5 Intervention/Usual Care

All participants received the usual standard of care at their participating hospital site. However, those randomised to the intervention also received the REDUCE intervention. This commenced with the ‘Initiation Phase’ consisting of eight one‐to‐one sessions delivered by an assigned facilitator (a HCP with specialist knowledge of diabetes, trained to deliver the REDUCE intervention using person‐centred facilitation skills and a cognitive‐behavioural approach). The sessions were structured to introduce and explore topics designed to promote skill development across the four target areas (i.e., regular foot‐checking; rapid self‐referral in the event of changes in foot‐health; graded and regular physical activity when ulcer‐free; and emotional/mood management). Together, these were intended to achieve the desired outcomes of reducing DFU recurrence and promoting healing if a DFU reoccurred. Content of individual sessions is summarised in Table 3.

**Table 3:** Intervention framework and techniques.

Engagement and self‐awareness (Sessions 1–2)		
Objectives	Interventions/Techniques	Change mechanism
Assessment and engagement Information giving Socialising to the CBT model Self‐awareness/Awareness of emotions Establishing baseline and targets	Empowering explanations Monitoring diaries and action plans Modelling foot checking Motivational techniques	Information about risk and health consequences Guidance on how to perform behaviours Increased self‐monitoring
**Active treatment (Sessions 3–6)**		
Embedding the model Management of emotions Using values to set goals Reviewing activities and building on successes Problem‐solving Recognising and overcoming obstacles Communicating needs Taking appropriate responsibility	Behavioural activation Behavioural experiments Activity scheduling Positive re‐framing Problem‐solving Barriers/Facilitators	Goal setting Action planning Monitoring thoughts and feelings and guided feedback Guidance on how to perform behaviours, including CBT techniques Positive feedback from behaviour changes
**Consolidation and handover (Sessions 7–8)**		
Building confidence Mood management Achievement of, or progress towards, REDUCE targets	Encourage socialising Establishing routine foot checking and care Establishing new routine activities	Relapse management Habit formation Self‐management

Alongside individual sessions, participants received a handbook to support their engagement and adherence. The handbook provided psycho‐educational exercises, reading materials and opportunities to record reflections, set personalised, values‐based goals and track progress.

During the ‘Initiation Phase’, participants also received unique login credentials to enable access to the DMI (as well as hard copies of the maintenance booklet). In the final face‐to‐face session, HCPs were instructed to show participants the DMI (if they had access to the internet) on a suitable device and provide guidance on how to use it to support long‐term behaviour change. Participants without internet access were advised how to use the booklets.

After completing the ‘Initiation Phase’, participants entered the ‘Maintenance Phase’ (i.e., self‐directed use of the DMI and/or maintenance booklet). Participants were also asked to opt in via the DMI to receive scheduled motivational emails designed to provide additional long‐term support for the four areas targeted by REDUCE, independent of DMI usage.

## 6 Results

### 6.1 Participants

One hundred and three patients were assessed for eligibility, of whom 74 were deemed eligible and were approached to participate. Twenty eligible participants agreed to participate and were recruited; 13 were randomised to receive REDUCE and 7 to usual care. Of these, 12 participated in baseline interviews and 9 in follow‐up interviews. A summary of participant characteristics is provided in Table 4.

**Table 4:** Participant characteristics.

	REDUCE (*n* = 13)	Usual Care (*n* = 7)	Overall (*n* = 20)
Male, *n* (%)	10 (76.9)	4 (57.1)	14 (70.0)
Age (years), mean (SD)	68.1 (11.0)	67.0 (15.3)	67.7 (12.3)
Ethnicity, *n* (%)			
White	12 (92.3)	7 (100)	1 (95.0)
Missing	1 (7.7)	0 (0)	1 (5.0)
Marital status, *n* (%)			
Married	8 (61.5)	5 (71.4)	13 (65.0)
Single, never married	1 (7.7)	1 (14.3)	2 (10.0)
Separated/divorced	1 (7.7)	0 (0.0)	1 (5.0)
Widowed	2 (15.4)	1 (14.3)	3 (15.0)
Co‐habiting	1 (7.7)	0 (0)	1 (5.0)
Highest level of education, *n* (%)			
School	7 (53.9)	0 (0)	7 (35.0)
College/sixth form	4 (30.8)	4 (57.1)	8 (40.0)
Undergraduate	2 (15.4)	2 (28.6)	4 (20.0)
Postgraduate	0 (0)	1 (14.3)	1 (5.0)
Employment status, *n* (%)			
Employed full‐time	2 (15.4)	1 (14.3)	3 (15.0)
Employed part‐time	1 (7.7)	0 (0)	1 (5.0)
Unable to work due to sickness or disability	1 (7.7)	2 (28.6)	3 (15.0)
Retired	9 (69.2)	4 (57.1)	13 (65.0)
Current smoker, *n* (%)	2 (15.4)	0 (0)	2 (10.0)
BMI (kg/m^2^), mean (SD)	33.1 (6.1)	32.4 (3.3)	33.9 (5.2)
Diabetes type, *n* (%)			
Type 1	2 (15.4)	2 (28.6)	4 (20.0)
Type 2	11 (84.6)	5 (71.4)	16 (80.0)
Most recent HbA1c, mmol/mol, median (IQR)	65 (52, 80)	59 (38, 75)	65 (51.5, 77.5)
Time since most recent ulcer (days), median (IQR)	19 (16, 23)	21 (14, 30)	19.5 (16, 25)

### 6.2 Results From Psychological and Behavioural PROMs

Questionnaires were returned by 16 (80%) participants at 6 weeks post‐randomisation and 15 participants (75%) at 3 months post‐randomisation. Results are shown in Table 5. While the small sample size precludes formal statistical comparisons, the trends in the data suggest that at 6 weeks, intervention participants reported improved footcare behaviour, better psychological well‐being, and more positive mood than the usual care arm. They also reported lower anxiety and depression, as well as lower levels of perceived fear, catastrophising and damage related to their diabetic foot disease. Levels of physical activity appeared to increase over time in the usual care arm and decline in REDUCE participants, although there was considerable variability between individuals on this measure. At 3 months, these broad patterns in the data remained, although attenuated. These findings align with the results from our first evaluation of the group‐based intervention [7], suggesting that changes to the format, delivery and content of the intervention did not diminish the therapeutic potential of REDUCE.

**Table 5:** PROMs at baseline and 6 weeks and 3 months post‐randomisation summarised by treatment group (means (SD) unless otherwise stated).

	Baseline		6 Weeks		3 Months	
	REDUCE	Usual care	REDUCE	Usual care	REDUCE	Usual care
Footcare Behaviour (NAFF)^a^	55.5 (8.1)	58.4 (6.8)	62.6 (6.3)	58.7 (7.1)	60.3 (8.7)	61.6 (6.0)
Illness Perception (BIPQ)^b^	32.5 (16.3)	37.4 (17.4)	44.4 (6.2)	46.0 (14.5)	37.4 (11.1)	35.6 (18.0)
Fear Avoidance (CBRQ‐FA)^b^	11.7 (4.5)	11.0 (3.4)	9.3 (6.0)	13.5 (4.2)	8.0 (4.0)	13.0 (5.5)
Catastrophisation (CBRQ‐C)^b^	7.0 (3.8)	6.7 (1.7)	4.6 (4.2)	7.6 (3.3)	6.1 (3.7)	4.4 (4.6)
Damage (CBRQ‐D)^b^	12.3 (3.0)	13.3 (3.1)	11.1 (2.5)	15.3 (2.6)	9.4 (4.3)	13.2 (3.9)
Positive Mood (SPANE‐P)^a^	23.4 (5.7)	20.9 (6.3)	22.1 (7.3)	20.0 (6.5)	20.8 (8.7)	21.6 (5.4)
Anxiety (GAD‐7)^b^	3.2 (5.9)	6.4 (6.5)	4.4 (6.2)	5.3 (5.9)	3.5 (7.1)	3.4 (5.0)
Depression (PHQ‐9)^b^	4.5 (5.9)	6.7 (6.4)	6.2 (8.5)	7.3 (5.9)	4.7 (8.0)	4.6 (4.2)
Mental Health (MHC‐SF)^a^	47.3 (19.8)	50.4 (15.5)	40.8 (20.3)	45.3 (18.0)	43.2 (19.3)	49.8 (16.9)
Physical Activity, MET‐Mins (IPAQ‐E‐SF)^a^	10,003.8 (9709.4)	6991.9 (11,181.9	7349.1 (8015.6)	15,843.2 (22,378.3)	6012.4 (5275.5)	13,207.2 (25,031.8)
Social Support (SPS)^a^	77.7 (15.1)	78.9 (10.7)	77.4 (10.6)	78.9 (11.3)	75.7 (14.4)	82.8 (8.1)

*Note:* An ‘a’ superscript indicates a higher score means a better state, while a ‘b’ superscript indicates a higher score represents a worse state.

### 6.3 Results From Nested Qualitative Study

#### 6.3.1 General Reactions

Participants' and facilitators' accounts provided a broadly consistent picture of a well‐conceived intervention that benefited from active tailoring. Most participants talked about the sessions in extremely positive terms and reported enjoying and looking forward to their conversations with facilitators. Both participants and facilitators highlighted the purposeful and productive nature of those interactions. Illustrative quotations are provided in Table 6.

**Table 6:** Illustrative quotations from a nested qualitative study.

Findings	Quotations
General reactions:	Finding sessions enjoyable… *‘It was pleasant to talk actually, because when you get to my age, and (are) mostly housebound, other than (at) the hospital … I don't see many people’ (Participant‐12U)* *‘Because I was isolated last year with the pandemic … I sort of looked forward to the phone calls, as much as doing the work for my feet…. I miss it a bit’ (Participant‐D6G)* *‘I think they actually all just liked the contact. They liked the talking … all said they enjoyed it, and I believed them’ (Facilitator‐019)* …and productive *‘We've talked about all sorts, it was brilliant…. Because I live on my own … having someone to talk to about fairly important things, through REDUCE, was great’ (Participant‐X1P)* *‘What is said in the phone call, helps you… not to go down the same route, or, (by) mak(ing) sure that you keep a check on things like … cracked skin … abrasions, or something like that. It helps you to understand why you have to do it, why it is needed’ (Participant‐K9H)* *‘We had lovely conversations, and I felt like … I'd certainly highlighted, or brought into focus, things that they hadn't thought of … things that they were going to change’ (Facilitator‐053)*
Individualised delivery:	Welcoming the one‐to‐one model *‘I prefer a one‐to‐one. I don't like talking in a big group, ‘cause I tend to get quite shy and I just sit back and listen. And when there's one‐to‐one, I think you can say what you feel, and what you want to do, with no‐one else listening, if you know what I mean?’ (Participant‐A4Z)* *‘With one‐to‐one, you can hit the stuff that's important, and stick at it, rather than it gets pushed to one side to talk about some other crap. That makes good sense to me. ‘Cause if they'd said, “This is all groups,” and not one‐on‐ones like we are now, then I'd've probably said, “No,” I wouldn't do it’ (Participant‐X1P)* Viewing intervention content as of variable *personal* relevance Foot‐care: *‘(Facilitator) got me to look at my feet in a different way … she got me to think more about my feet, and it got me to start checking them every day, which previously I hadn't done’ (Participant‐D6G)* Physical activity: *‘Part of my retirement plan (was) to do walking, activity … so I was already down that activity track. What it probably has done is made me think more about that activity, what I'm doing and the impact on my feet’ (Participant‐L5E)* Help‐seeking: *‘In the past, if I've ever had a problem with my toes or my foot, I've gone straight onto the clinic and I've been seen within a day or two. I've never allowed anything to go on longer than that, they've always been very good. So, I'm aware of that and I've always done that, so that has never been a problem’ (Participant‐V11)* Low mood: *‘There were … discussions about mental attitudes … I have a very positive outlook on life, so that isn't something that affects me. (But) I think it could affect someone, and in fact I think it could affect someone quite severely’ (Participant‐F3W)* Similarly mixed responses to the therapeutic approach ‘Think‐Feel‐Do’ model: *‘The Think Feel Do approach, you know, that's definitely part of my psyche now’ (Participant‐L5E)* *‘In everyday life, you just sort of get on with things, and you don't often think “Oh, how do I feel about this?” because you've got sometimes quite a bit to do!’ (Participant‐D6G)* Goal‐setting: *‘Writing stuff down … the values, and the objectives, and goals … sounds silly, I know, but that had a really big impact on me…. To actually write that down, and say well, “How am I going to do that?” And to set a target … it was quite rewarding to say, I set myself some targets, and I either met them or exceeded them. It was really motivating for me’ (Participant‐L5E)* Diary‐keeping: *‘Once the diary had been going for a while, you could look back and see that you were improving, you know … (and) once you can see a track, track record, you know, and it's improvement, it gives you an incentive, doesn't it?’ (Participant‐M10)* *‘That diary was difficult because, obviously, I'm busy during the daytimes … I'm doing things, and the diary, I'd come back and think, Oh God, what was I doing at 10 o'clock? And I found that a bind to keep doing that. So, I gave up on it, I told (Facilitator), it was too much like homework’ (Participant‐V11)* Hence, some tailoring was essential *‘(The) gentlemen—weren't so mobile, I explained they didn't have to do everything … I said, “Well, you could do X sitting”’ (Facilitator‐022)* *‘How it's pitched, was quite important … it fitted different people in different ways…. And I think it does need some … tailoring … that's what I hope I did’ (Facilitator‐019)*
Remote delivery:	Judging this an acceptable (often preferable) model *‘When we all started to use Zoom … I thought I … much preferred the face‐to‐face thing. But … it saves so much time, and travel … I think it's probably going to be the way forward now, to be honest’ (Participant‐I8C)* *‘From home, on the phone, I'm better at that, because … sometimes I get a bit, what do you call it, tongue‐tied? And when that happens, I just, I panic, and then, well, it'd be the end of the session’ (Participant‐ K9H)*
Intervention resources, the DMI and maintaining change:	Mixed independent use of intervention resources, for example, handbook… *‘(One) obviously read the handbook before the sessions, and he would have it there … (the others) were pretty, a bit more chaotic’ (Facilitator‐53)* *‘I used (the handbook) sometimes outside of sessions, in terms of, basically I'd always look at it when we're going to get together, I'd always have a look to see what we're probably going to talk about, and I would, I would use the same opportunity to kind of revise what we've spoken about the week before … I've also, and I can't think what now, but I have referred back to it’ (Participant‐I8C)* *‘I did initially use them, but then when I got to the one‐to‐one with (Facilitator), I'd pretty well sussed out what was required’ (Participant‐M10)* …and very limited use of the DMI *‘(Facilitator) did mention (the DMI). And she did, I think she sent me an email with a link to it … like a complete idiot, I totally forgot about it … (and) there's a very good chance that I have lost it’ (Participant‐X1P)* *‘(Facilitator) sent me the link, and I did actually do it with her on the last session, I logged in and that, and then she sent me the, how can I say, the path, or the link, but I lost the email, so I haven't been able been able to log in (since)’ (Participant‐M10)* Expressing varying confidence about maintaining behavioural changes *‘I am quite confident that I will be able to do it, because … I have walked regularly, for many, many years … and I've always, always enjoyed it…. So, once I'm on a roll, I'm pretty okay with it, and … I really do understand, that it kind of makes me feel better … that kind of behaviour is now kind of implanted in my mind, so I don't have to be reminded or encouraged’ (Participant‐I8C)* *‘My family, they've been through the programme (too) … (Facilitator) had two weeks (away) … and in that two weeks, my family (said), “We have to do this, you cannot stop!”’ (Participant‐K9H)* *‘I know what I'm like, right. I can get into a good routine … where everything's going along swimmingly. And then six months down the line, something happens … and I'll fall off the horse’ (Participant‐X1P)* *‘At present my life is lovely, everything is going well, and I'm very fortunate, but if it was to go wrong in any way, then obviously my mind will be in a different area, and these things might not be as important as they were before’ (Participant‐V11)*

#### 6.3.2 Individualised Delivery

Participants often cited the one‐to‐one format of the intervention as a key factor in their decision to enrol in the pilot trial. Many described how they had welcomed and benefited from the opportunity to converse with an empathetic listener about a wide range of concerns. Participants and facilitators emphasised how the one‐to‐one nature of the intervention had enabled them to explore personal problems and barriers to behaviour change and to work together to come up with bespoke strategies or solutions. Both participants and facilitators also highlighted the varying personal relevance of the core intervention content. For example, while participants said they had expected foot care to be part of the ‘curriculum,’ and almost all reported new learning (changes in both thinking and practice) in this area, many varied in their views on the personal salience of the other core topics (physical activity, help‐seeking and managing low mood). This perspective was sometimes shared by facilitators, who described how they sought to tailor and direct the intervention to reflect individuals' histories, circumstances and needs. Participants acknowledged, however, that content of less immediate relevance to them might have value to others.

Participants and facilitators also described varied responses to different elements of the therapeutic approach. While some participants reported finding the central ‘Think‐Feel‐Do’ model helpful, others appeared to struggle with it or did not recall the model at all. Participants expressed similarly mixed perspectives on ‘goal setting’, with this technique appearing to resonate more strongly with younger participants (under 70 years). Facilitators described considerable difficulties engaging some older participants with the concept of working towards personal goals and reported adjusting their language accordingly (for instance, talking about ‘making plans’ instead). Self‐monitoring similarly elicited a range of responses. Some participants said they had found the use of ‘diaries’ to record their activities interesting and motivational, but others reported swiftly abandoning this task, finding it onerous or inconvenient.

#### 6.3.3 Remote Delivery

Participants generally talked favourably about remote delivery, viewing this as acceptable and, in many cases, preferable to in‐person sessions, which would have required travel to appointments. They highlighted the benefits of a reduced risk of Covid‐19 infection, convenience and comfort. Approximately half of the participants had opted to take part in sessions by telephone (as opposed to video call). Facilitators who delivered telephone sessions highlighted differences and additional challenges associated with that format and observed that intervention materials assumed participants and facilitators could see each other.

#### 6.3.4 Intervention Resources, the DMI and Maintaining Change

Facilitators regarded the patient handbook supporting the ‘Initiation Phase’ sessions as a valuable resource, which they used to help anchor their conversations. However, they also indicated that participants' use of this, particularly outside of sessions, was mixed. Participants' own accounts supported this perception. Some said they used the handbook between sessions and to prepare for them. Others described giving it a much more cursory look. Regarding the DMI, accounts suggested limited independent use. Many participants (around half) did not use the internet at all. Of those who did, several reported not having accessed the DMI, having forgotten about it or having lost their login information. Only a couple of participants reported having used the DMI independently by the time of their follow‐up interview. Those who did said they judged it as offering ‘nothing new’ and did not (at that time) plan to revisit it.

Nevertheless, participants recognised the importance and challenge of sustaining the new practices and routines they had established via REDUCE and varied considerably in their confidence that they would be able to do this without further longer‐term facilitator support. Some expressed optimism that they now had the tools, motivation and familial support or pressure they would need to sustain changes. In contrast, others reported already diminishing motivation or surmised that future life developments might make footcare less of a priority.

### 6.4 Intervention Recommendations

In line with previous approaches to refining complex interventions, we adopted a structured approach for translating findings from the pilot trial into actionable refinements to REDUCE [37]. This involved the formation of ‘Collaborative Working Groups’ comprising investigators and researchers involved in all aspects of the REDUCE intervention, including the DMI, and the qualitative and quantitative evaluations conducted during the pilot trial. Over successive meetings, the group used the ‘What? So what? Now what?’ framework [38] to identify actionable issues. Tables 7 and 8 summarise these issues and the resulting changes to the REDUCE intervention.

**Table 7:** Modifications to the initiation phase.

Issue/problem/change identified (‘What?’)	Why the issue/problem/change is required/important (‘So what?’)	Description of change/amendment required (‘Now what?’)
Goal‐setting language The language and idea of setting goals and working towards these outside sessions appeared to resonate much more strongly with younger participants (under 70 years). In contrast, older participants (in their 70s and 80s) had difficulties recalling and recounting goals they had set: some were described by HCP as struggling with the idea of setting and working towards personal goals and/or being resistant to change.	*May discourage engagement with key behaviour change aspects of the intervention in some participants as it stands*.	Changes to materials Reduce the number of goal‐setting sheets. Update terminology in the handbook and sheets to provide alternatives. For example: Goals (things I plan to do in the week) Remove ‘homework’ phrase and update. Changes to HCP intervention training Greater focus on training for HCP to work with participants to review written goals, reflect and revisit at the next session. Update the HCP training manual to reflect this. Greater focus on training in personalising and individual approaches tailored to participants' needs/lives. Support experience and confidence in being fluid with the language.
Engagement with self‐monitoring Some participants reported finding diary writing interesting and powerful, though this was rarely sustained throughout the programme. A few participants reported that they had found diary‐keeping difficult and/or inconvenient and swiftly abandoned it.	*May discourage engagement with key behaviour change aspects of the intervention in some participants as it stands*.	Changes to materials Simplify and reduce the overall number of diary sheets Emphasise the benefits of diaries in the participant handbook Changes to HCP intervention training Greater focus on supporting HCPs to be selective about diary use and focus on certain aspects relevant to the individual. Additional training and practice were provided on introducing self‐monitoring/diaries to participants for the first time.
Variable use of the participant handbook HCP facilitators described their sense that participants varied in the use of the participant handbook. In interviews, patients reported a range of uses: some very limited use of the manual (giving it an initial ‘thumb through’ only); others said they had used it to prepare themselves for sessions and/or to ‘revise’ what they had discussed previously. Some reported that the handbook could have had some modifications to be more useful.	*Participant handbook seen as a valuable resource for participants and HCPs throughout the initiation phase and potentially beyond. It acts as additional scaffolding to support the sessions. Ideally needs to accommodate different needs and uses to maximise benefit*.	Changes to materials Make some adaptions/tweaks to the participant handbook to reflect the different uses described by participants to ensure key messages are communicated. Include a mini‐series/synopsis of what to expect in each session at the beginning of the handbook, and for each session section in the handbook. Include a box of helpful things to recap before next session (e.g., planning for next week). Update participant intervention materials letter sent out with participant handbook to clarify rationale. Changes to HCP intervention training Add additional emphasis to train HCPs to individualise how the materials are used with the participant.
Certain topics seen as of no personal relevance Some topics/areas targeted by REDUCE (help‐seeking, low mood and physical activity) were viewed as not relevant for them individually. Participants indicated that discussing these topics was not a good use of their time.	*While this was expected, the extent of this feedback indicated there might be scope and benefits to more explicitly acknowledging the varying relevance of the REDUCE topics in participant materials.*	Changes to materials Add notes in participant handbook and maintenance booklet(s) to confirm these may not be relevant to you/are only relevant to certain people. Make clearer in the participant handbook why each topic is included in REDUCE, recurrence can happen, and REDUCE is preparing participants for this, as it may be relevant in the future. Changes to HCP intervention training Additional emphasis to be placed on sensitising HCPs to the variation of participants they will encounter and how to tailor REDUCE effectively. Both considering the immediate—and things that might be relevant for the future. Important not to diminish their importance. Add training on shifting attention when needed.
Lack of knowledge of the REDUCE model Some participants reported finding the Think‐Feel‐Do model helpful, but others seemed to have found it less so, and several did not appear to have any recollection of it.	*This model is considered core to the REDUCE intervention and all participants need to be at least familiar with it by the end of the programme*.	Changes to materials Give the model more prominence throughout participant materials Changes to HCP intervention training Emphasise the importance of the model and the need to communicate this to participants, as considered core to the REDUCE intervention

**Table 8:** Changes to the maintenance phase.

Issue/problem/change identified (‘What?’)	Why the issue/problem/change is required/important (‘So what?’)	Description of change/amendment required (‘Now what?’)
Diminishing motivation to maintain changes post‐sessions Some participants indicated that changes in their wider lives after REDUCE might make their newly learned practices less of a priority and/or reported that after their final one‐to‐one session, their motivation, had (already) started to drop.	*REDUCE seeks to make behavioural changes that are maintained to help participants over the longer term once sessions are over. Participants should be encouraged to think about how they will maintain motivation/changes once the intervention ends*	Changes to materials Maintenance of changes to be given greater prominence in participant handbook—with content relating to this from Session 2 onwards Greater emphasis to be added on exploring the digital and offline maintenance materials before the sessions end in the participant handbook Changes to HCP intervention training Greater emphasis on introducing the maintenance materials earlier in the programme Greater emphasis on encouraging participants to think about how they can use the available tools and materials to support them once sessions end.
Low engagement with maintenance materials, particularly the website There was variation between HCPs, and participants, with regard to whether and how the maintenance intervention had been introduced. Levels of independent engagement with the digital website were low, with participant interviewees (who used the internet) saying they had not got round to it; had forgotten about the website; had lost the link/log‐in information; and/or accessed it but judged it as offering them ‘nothing new’ (i.e., not warranting extended attention or further visits).	*Maintenance of changes is core to the REDUCE aims. Feedback and usage raise questions about maintenance materials, their integration with the programme and effective introduction/promotion by facilitators*	Changes to materials Increased visibility of access details of digital maintenance website (dedicated space in participant handbook, include in cover letter) Highlight additional content covered in the maintenance website and booklets Include digital maintenance website access and usage guide in both the participant handbook and maintenance booklet Change email motivational messages to be opt‐out rather than opt‐in Create truncated text versions of motivational emails for participants unable or unwilling to access the website, to be automatically sent at regular intervals during follow‐up Changes to HCP intervention training Increased ‘hands‐on’ training on using the digital maintenance website to enable support and early demonstrations via screen sharing to participants. Greater focus on communicating the value and creating excitement regarding the maintenance support, particularly the digital maintenance website for those with the capacity to access

## 7 Discussion

**Figure 3:**
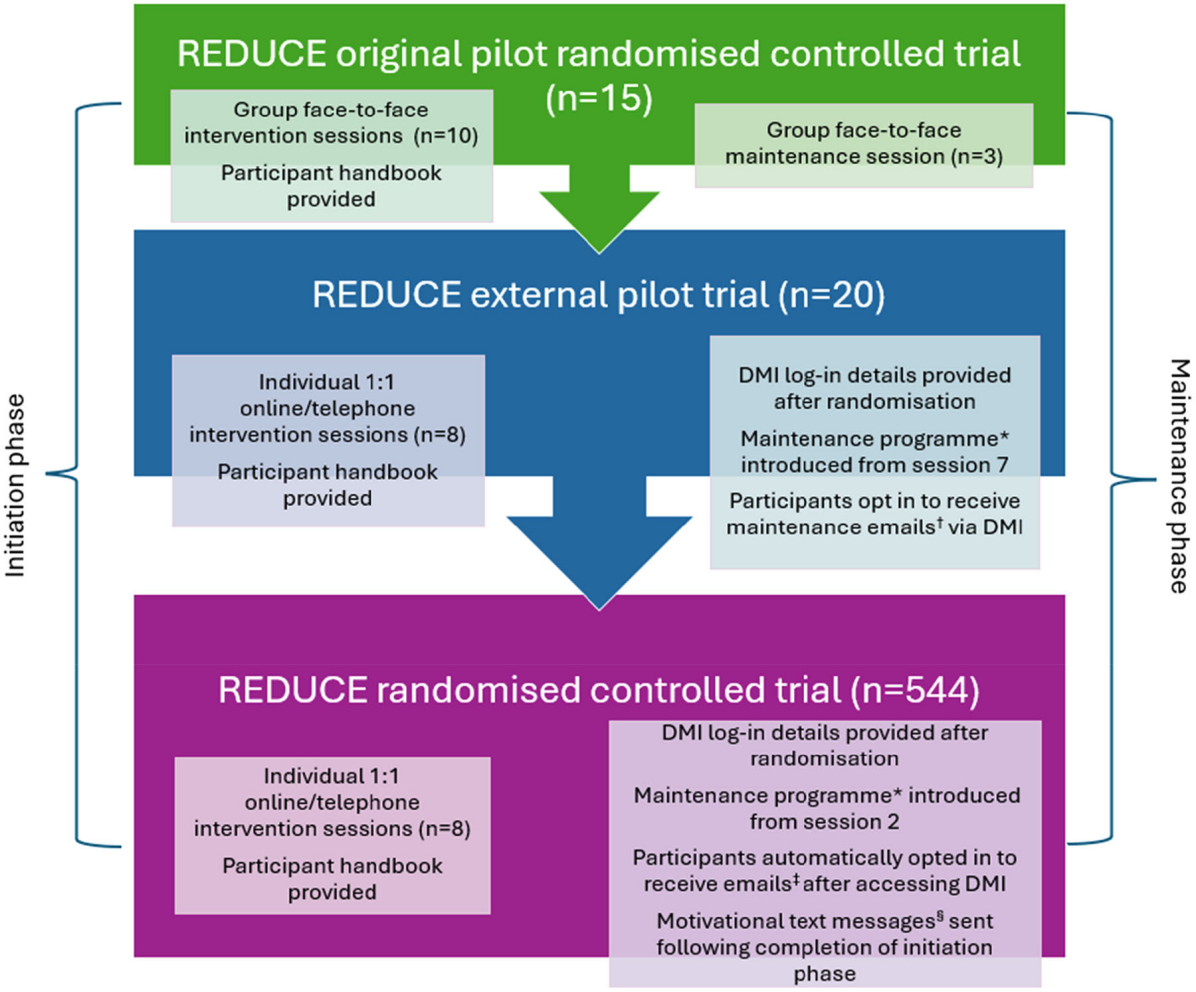
REDUCE intervention refinement. * Maintenance programme included hard‐copy booklets (In the pilot trial these were 5 separate short booklets, ‘Checking Your Feet’ and ‘Strength and Balance’ sent before Session 1, ‘Improving Your Mood’, ‘Getting Active’ and ‘Getting Help Quickly’ sent before Session 6; in the main trial these were combined in a single handbook and sent after Session 2) and the digital maintenance intervention (website). † 8 weekly emails and 5 bi‐weekly emails (*n* = 13 over 18 weeks). ‡ 8 weekly emails and 35 bi‐weekly emails (*n* = 43 over 79 weeks) from the point of accessing the DMI. § Weekly motivational texts (*n* = 42 over 41 weeks) from the week following completion of the initiation phase.

We have reported here on the iterative multi‐method, multidisciplinary and collaborative process that we engaged in to develop the REDUCE intervention. A summary of the main stages of the intervention's evolution, including the final version that is currently the subject of an effectiveness and cost‐effectiveness trial, is provided in Figure 3.

Our first aim here was to formally record the process of developing the intervention, as this information will be crucial to determining, in accordance with established guidance, whether REDUCE worked as intended, for whom and in what contexts [39]. However, the details provided here also guard against the risk, particularly during long programmes of research, of historical knowledge being eroded with time. The development of complex interventions could be regarded as particularly susceptible to such a challenge, given that they are necessarily made up of many ‘moving parts’ which evolve dynamically and iteratively over time [40]. Failure to document how the intervention has evolved could result in inadvertently returning to versions of the intervention previously discounted for valid reasons. For example, REDUCE began as a group‐based programme. However, in advance of the pilot, it was reconfigured to be delivered one‐to‐one. Our findings reveal that not only was this format well received, but it also afforded opportunities to individualise the intervention, which we can hypothesise may enhance its effectiveness. It is conceivable that, without the historical overview presented here, stakeholders considering the implementation of REDUCE in the future might inadvertently advocate for a group format without recognising the potential implications for effectiveness.

Our second aim was to share broader learnings regarding the development of complex behavioural interventions in health, which we hope will benefit others working in this arena. The first relates to the considerable added value of embracing multiple methods and disciplinary perspectives. The work described here covers diverse, but complementary methods that enabled us to: examine how participants engaged with the digital aspects of the intervention (think‐aloud methods); explore the experiences of HCPs delivering the intervention and participants in receiving it (qualitative interviews); identify early signs of intervention effects (pilot RCT); and determine how to prioritise areas for refinement (person‐based approach). Although REDUCE is, at its core, a cognitive‐behavioural intervention, we have drawn on a range of social and behavioural sciences during its development (health psychology, human factors, sociology, cognitive behavioural therapy and project management), underscoring the importance of multi‐disciplinarity in health research and the integration of methods as advocated by the World Health Organisation [41].

A second and related issue concerns the role of PPIE in the development of the intervention. The central role of PPIE in health research is now well‐established internationally [42], and there is growing recognition of how it can complement formal qualitative research with study participants [43]. This was clearly demonstrated in the development of REDUCE. For example, our PPIE group was formed several years before we commenced Stage 1 of the research. This meant that when the Covid‐19 pandemic made recruitment for non‐Covid‐related research impossible, we were still able to commence work on optimising the DMI by conducting this study with members of our PPIE panel. We acknowledge that the scale of the work was not as we had intended (we had originally planned to conduct think‐aloud interviews with 20 participants), and our findings may consequently have been less robust. However, we were subsequently able to prioritise examining the use of the DMI in our Stage 2 qualitative interviews with trial participants. Similarly, we were able to consult our PPIE panel on unplanned innovations to the intervention that arose out of the pilot trial, such as the introduction of weekly text messages. PPIE allowed us to assess the acceptability of both message content and frequency in advance of their implementation in the trial. A summary of how PPIE was involved in all stages of the research described here is provided in accordance with GRIPP2 [44] guidance in Appendix 1.

A third observation concerns the promise of digital and remote interventions in health. REDUCE embodies both in that the Initiation sessions were all delivered remotely (via telephone or online) and the Maintenance features of the intervention were web‐based (although printed maintenance booklets were also made available). With regard to the former, our pilot trial findings highlighted that remote delivery was largely welcomed by our participants, and no clear disadvantages were reported by our facilitators or in PROM responses. This is in keeping with a growing body of evidence suggesting that while patient satisfaction is often ill‐defined in this literature, overall, patients appear to report reasonable satisfaction with remotely delivered interventions [45].

In contrast, the issue of satisfaction with digital interventions is less clear. In the United Kingdom, the widespread implementation of digital technologies is one of the central pillars of the NHS Long‐Term Plan and has also been advocated in the 2024 independent investigation into the NHS [46]. The explicit aim of such technologies is to enable health services to deliver more effective and efficient services. Indeed, this resonates with our own motivation to adapt our original Maintenance sessions into a web‐based digital support package. However, our pilot trial highlighted that while many participants recognised the need for long‐term support, very few appeared to use the DMI for this purpose. It is unclear at this point whether this is a function of also providing participants with hard‐copy maintenance booklets, which may have served to make the DMI redundant, or whether there are additional challenges associated with engagement with digital technologies for this patient group that we must address. Digital technologies are, of course, not new in diabetes, and some are supported by strong evidence of satisfaction and effectiveness [47]. However, it may be appropriate to contrast the REDUCE DMI with other technologies, such as continuous glucose monitoring or telehealth. The REDUCE DMI differs from these in that usage and engagement are determined solely by the patient and are not linked to scheduled goals or objectives (e.g., planned clinical reviews and maintaining glucose levels within specific ranges). Although the REDUCE DMI is supported by regular emails and text messages designed to direct patients to use it, ultimately, patients choose whether and how to engage with it (see Table 8). We will have to await the findings of the effectiveness trial to determine if these have increased engagement with this aspect of the intervention.

Finally, we would also like to acknowledge some potential limitations. First, as noted above, the scale of our think‐aloud study in Stage 1 was significantly reduced due to the pandemic. Second, the timing of the follow‐up interviews in Stage 2 precluded us from exploring participants' long‐term use of the DMI and so we were unable to determine whether modifications would be required to sustain engagement. Third, limitations in funding and time prevented us from implementing all the refinements to the intervention that were highlighted by the think‐aloud and pilot trial. However, we have sought to be systematic and transparent in documenting which modifications were made and which were not.

In conclusion, REDUCE is a complex behavioural intervention addressing a complex health problem—namely, reducing the risks of re‐ulceration and prolonged ulcer healing in people with diabetes. We have reported here on the multidisciplinary and iterative approach we have taken in developing the intervention and have endeavoured to provide a detailed account of the strengths and limitations of our approach.

## Author Contributions

Conceptualisation: Kavita Vedhara, Fran Game, Kat Bradbury, Julia Lawton and Trudie Chalder. Methodology: Kavita Vedhara, Debbie Brewin, Christina Sheehan, Kieran Ayling, Kat Bradbury, Ruth Hart, Natasha Mitchell and Trudie Chalder. Data curation: Debbie Brewin, Christina Sheehan, Kieran Ayling and Ruth Hart. Investigation: Debbie Brewin, Christina Sheehan, Kieran Ayling, Joanna Slodkowska‐Barabasz, Judith Joseph and Ruth Hart. Formal analysis: Kieran Ayling and Ruth Hart. Supervision: Kavita Vedhara, Fran Game, Kat Bradbury, Julia Lawton and Trudie Chalder. Funding acquisition: Kavita Vedhara, Fran Game, Kat Bradbury, Julia Lawton and Trudie Chalder. Project administration: Christina Sheehan, Kieran Ayling, Ruth Hart and Natasha Mitchell. Writing – original draft: Kavita Vedhara. Writing – review and editing: Debbie Brewin, Fran Game, Christina Sheehan, Kieran Ayling, Kat Bradbury, Joanna Slodkowska‐Barabasz, Judith Joseph, Ruth Hart, Natasha Mitchell, Julia Lawton and Trudie Chalder.

## Disclosure

The views expressed are those of the author(s) and not necessarily those of the NIHR or the Department of Health and Social Care.

## Ethics Statement

The authors have abided by the Ethical Principles of Psychologists and Code of Conduct as set out by the BABCP and BPS.

## Consent

Any necessary informed consent to participate/for the results to be published has been obtained.

## Conflicts of Interest

The authors have no conflicts of interest to declare.

## Data Availability

The data that support the findings of this study are available on request from the corresponding author, K.V.

## Appendix 1

See Table A1.

**Table A1:** Patient and Public Involvement and Engagement (PPIE) activities in the optimisation of the REDUCE intervention and the REDUCE pilot trial (based on GRIPP2 short form).

Aims of PPIE	Methods	Findings	Impact	Other considerations
Optimise the acceptability of all patient‐facing materials and the participant interview topic guide for use in the REDUCE external pilot trial.	PPIE review of participant information sheet, consent form, contact details form, REDUCE participant and maintenance handbooks and the participant interview topic guide. Review of the patient‐related outcome measures (psychological and resource use questionnaires). Testing confirmed the time required to complete the baseline and 6‐week and 3‐month questionnaires.	Typographical amendments made to patient‐facing documents. Only minor modifications were suggested regarding the use of the term ‘homework’, which may have negative associations; therefore, ‘home practice’ was recommended. Instructions added to front of questionnaires confirming duration to complete (approximately 20 min for 6‐week and 45 min each for baseline and 3‐month).	Implemented recommendations led to improved patient‐facing documents and materials, which may support recruitment and retention in the REDUCE RCT.	
Update the DMI user guide for the REDUCE randomised controlled trial (RCT) and identify ways to improve accessibility and use.	The DMI user guide was tested by PPIE alongside accessing and using the DMI. Accessibility and use of the DMI in the external pilot trial was discussed and ways to increase access identified for the REDUCE RCT.	Feedback addressed included clarity of wording, typographical amendments and inclusion of additional screenshots to demonstrate the webpage and action required in the user guide. Reminders to access the DMI and the introduction of the DMI by the facilitator earlier in the REDUCE intervention sessions. Provision of emails with specific, individual‐tailored content.	Clear DMI user guide, appropriately tested. Reminders and earlier access and discussion about the DMI may support increased access to the DMI in the REDUCE RCT. Generic emails included within the DMI.	Individual‐tailored email content is not within the research budget. Potential to explore in future research.
Identify lessons learned from the REDUCE external pilot trial and improvements for the REDUCE randomised controlled trial (RCT)	The PPI group identified and discussed reasons why patients declined to participate and how this could be improved for the RCT. Reviewed and provided feedback on the motivational text messages to be used in the REDUCE RCT. Feedback included using hyperlinks or QR codes.	Identified that an infographic might be useful to present a study summary before reading the participant information sheet. The duration to complete questionnaires may be a factor in participation; therefore, the questionnaire length was reduced. Feedback included using hyperlinks or QR codes within the text message.	Infographic developed with and reviewed by PPIE for use in the REDUCE RCT. Questionnaires reduced in length with items removed and short‐form scales used where appropriate following discussion with wider team about outcomes. Requested clarification about the term ‘diabetic foot disease’. The latter was not amended, as the 20 participants in the pilot trial did not report any issues relating to the use of this term. Hyperlinks included within the text messages and emails sent in the REDUCT RCT	
